# Supplementary material for: Human Papillomavirus 16 E7 Promotes EGFR/PI3K/AKT1/NRF2 Signaling Pathway Contributing to PIR/NF-κB Activation in Oral Cancer Cells
Source: Cancers (Basel). 2020 Jul 15;12(7):1904. doi: 10.3390/cancers12071904 (PMC7409273; doi:10.3390/cancers12071904)
Supplement: Supplementary file 1 [file cancers-12-01904-s001.pdf]

Supplementary Materials

Human Papillomavirus 16 E7 promotes EGFR/PI3K/AKT1/NRF2 signaling pathway contributing to PIR/NF-κB activation in oral cancer cells

Diego Carrillo-Beltrán, Juan P. Muñoz, Nahir Guerrero-Vásquez, Rancés Blanco, Oscar León, Vanesca de Souza Lino, Julio C. Tapia, Edio Maldonado, Karen Dubois-Camacho, Marcela A. Hermoso, Alejandro H. Corvalán, Gloria M. Calaf, Enrique Boccardo and Francisco Aguayo

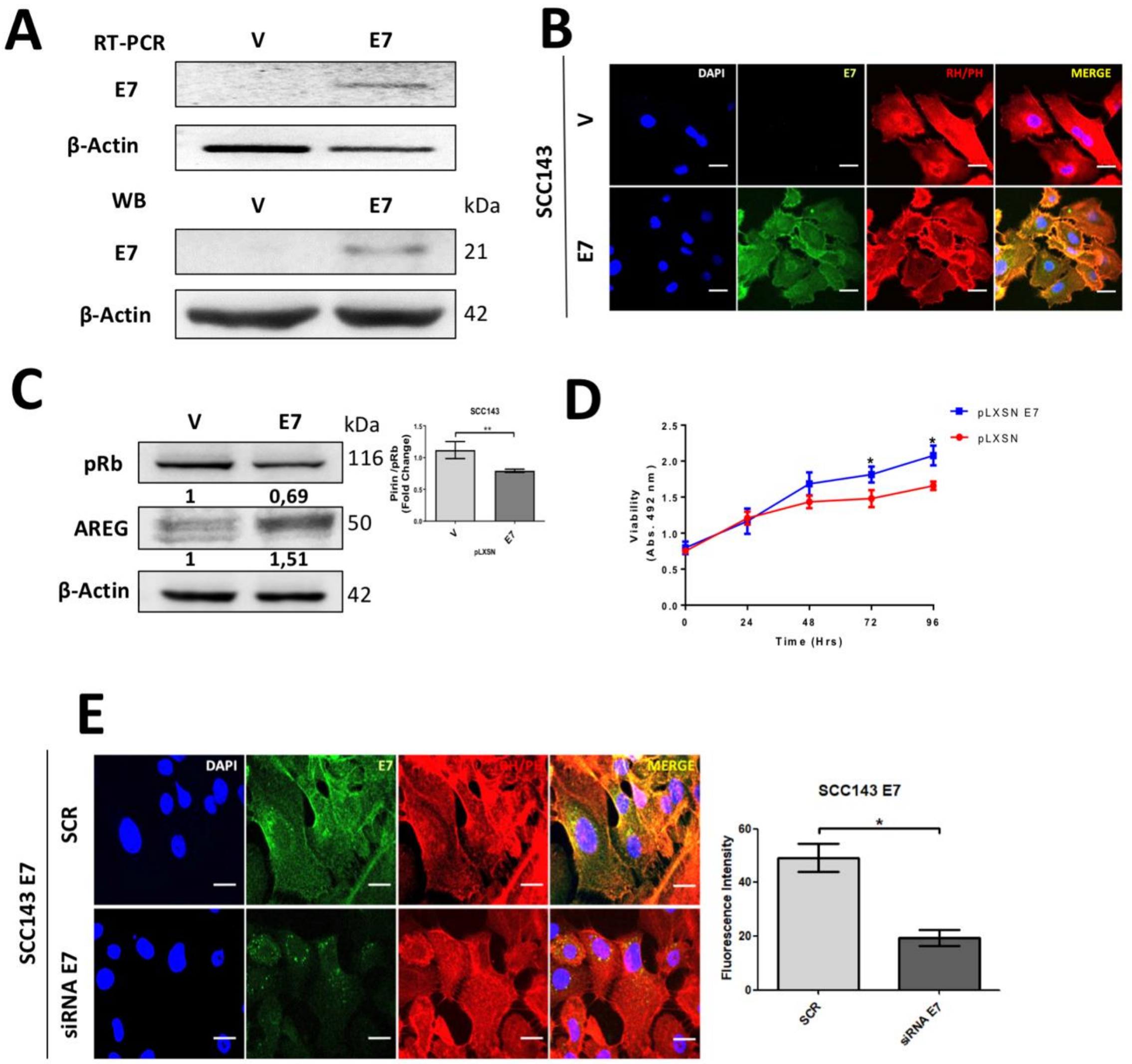

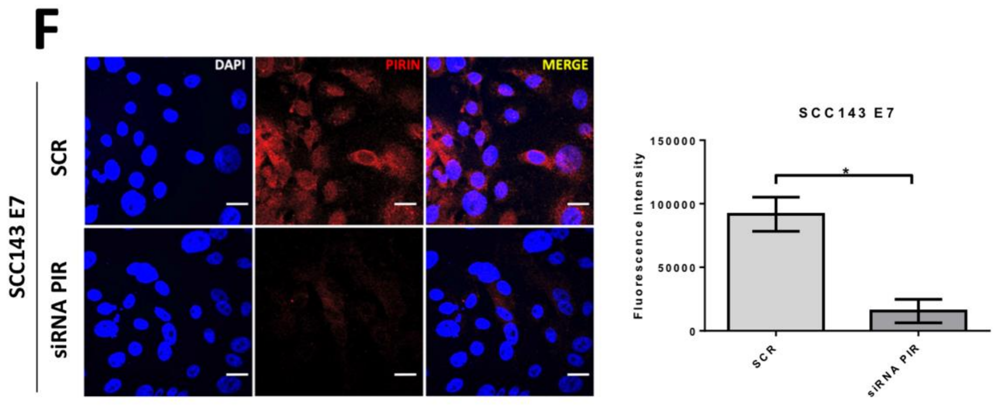

Figure S1. Expression and functionality of E7 in oral cells. (**A**) RT-PCR and WB were performed to check transcripts and protein levels of E7 and β-actin, respectively. (**B**) IFI reveals E7 expression in SCC143/E7 cells and not in SCC143/V cells, scale bar: 5 µm. (**C**) WB to evaluate the levels of AREG and pRb protein in SCC143/E7 and SCC143/V cells using β-actin as a load control. The graph represents densitometric analysis of three independent WBs for pRb protein normalized by β-actin. (**D**) Viability test with MTS in SCC143/E7 and SCC143/V cells from 0 to 96 h post-sowing. (**E**) IFI to detect E7 protein in SCC143/E7 cells previously transfected with random sequence siRNA (SCR) and siRNA E7. Scale bar: 5 µm. (**F**) IFI to detect Pirin protein in SCC143/E7 cells previously transfected with random sequence siRNA (SCR) and siRNA *PIR*. Scale bar: 5 µm. Data are presented as the mean ± SEM; average of three independent experiments, conducted in triplicate.* $p < 0.05$ (Mann-Whitney test).

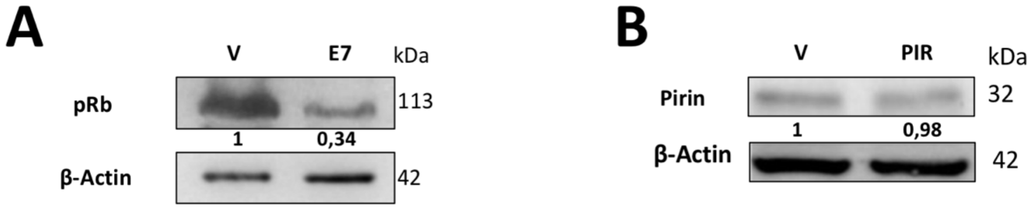

Figure S2. Phenotypic changes associated with HPV16 E7 and Pirin levels in SCC143 cells. (**A**) WB to evaluate levels of pRb protein in OKF6-TERT2 V and E7 oral organotypic raft culture cells. (**B**) WB in SCC143 V (pcDNA 3.1-eGFP) and SCC143 PIR (pcDNA 3.1-eGFP-PIR) cells to evaluate levels of Pirin by using β-actin as a load control. Data are presented as the mean ± SEM; average of three independent experiments, conducted in triplicate. * $p < 0.05$ (Mann-Whitney test).

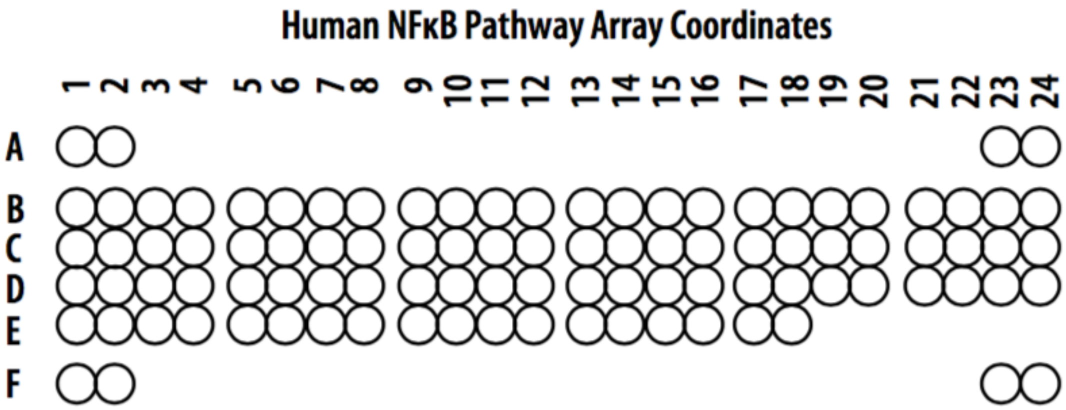

Coordinate	Target/Control	Entrez Gene ID	Alternate Nomenclature
A1, A2, A23, A24	Reference Spots	NA	RS
B1, B2	ASC	29108	PYCARD/CARD5
B3, B4	BCL10	8915	CLAP/CIPER/CARMEN/c-E10
B5, B6	CARD6	84674	CINCIN1
B7, B8	CD40/TNFRSF5	958	CDW40
B9, B10	cIAP1/BIRC2	329	———
B11, B12	cIAP2/BIRC3	330	———
B13, B14	FADD/MORT1	8772	GIG3
B15, B16	Fas/TNFRSF6/CD95	355	———
B17, B18	IκBα	4792	NFKBIA/MAD-3/NFKBI/IKBA
B19, B20	IκBε	4794	NFKBIE/IKBE
B21, B22	IKK1/IKKα/CHUK	1147	IKKA/IKBKA/TCF16
B23, B24	IKK2/IKKβ	3551	IKKB/NFKBIKB/IMD15
C1, C2	IKKγ/NEMO	8517	IKBKG/FIP3/IMD33
C3, C4	IL-1 RI	3554	CD121A/IL-1R-alpha
C5, C6	IL-17 RA	23765	CD217/CANDF5/CDw217
C7, C8	IL-18 Rα	8809	CD218a/IL18RA/IL1 RRP
C9, C10	IRAK1	3654	IRAK/pelle
C11, C12	IRF5	3663	SLEB10
C13, C14	IRF8	3394	ICSBP/IMD32A
C15, C16	JNK1/2	5599/5601	SAPK1/MAPK8; SAPK1a/MAPK9
C17, C18	JNK2	5601	SAPK1a/MAPK9
C19, C20	LTBR/TNFRSF3	4055	CD18
C21, C22	Metadherin/AEG-1	92140	LYRIC/MTDH
C23, C24	MYD88	4615	———
D1, D2	NFκB1	4790	p50/p105
D3, D4	NFκB2	4791	p52/p100
D5, D6	NGF R/TNFRSF16	4804	CD271/p75NTR
D7, D8	p53	7157	———
D9, D10	p53 (pS46)	7157	———
D11, D12	RelA/p65	5970	NFKB3
D13, D14	RelA/p65 (pS529)	5970	NFKB4
D15, D16	c-Rel	5966	———
D17, D18	SHARPIN	81858	SIPL1
D19, D20	SOCS6	9306	CIS4/SSI4/SOCS4/STATI4
D21, D22	STAT1p91	6772	ISGF-3/STAT91
D23, D24	STAT1 (pY701)	6772	ISGF-3/STAT91

Figure S3. Human NF-κB Pathway Array Coordinates.

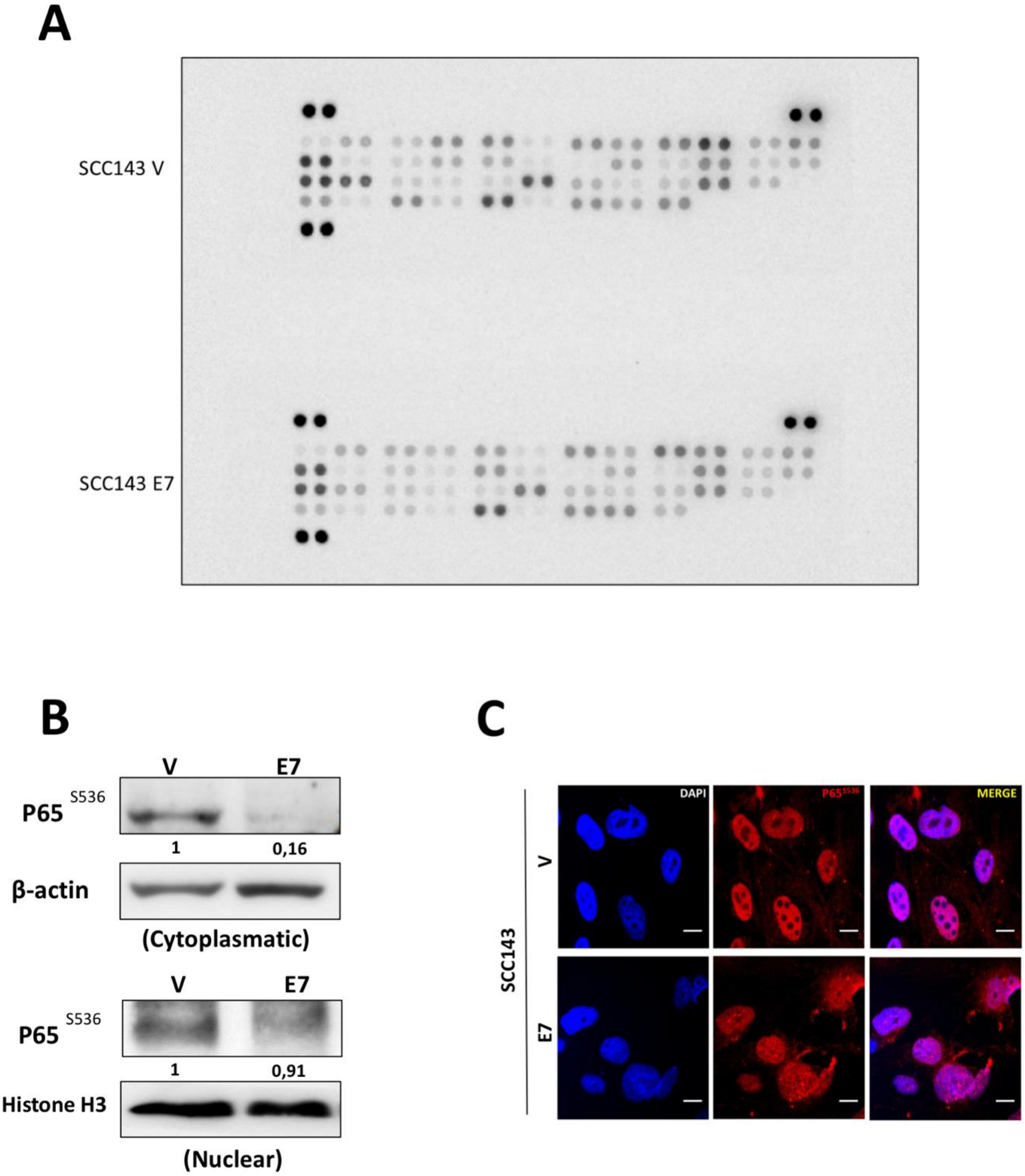

Figure S4. HPV16 E7 expression induces changes in NF-κB pathway in oral cells. (**A**) Proteome Profiler Array NF-κB in conditions V and E7 exposed 250 seconds (**B**) WB of nuclear and cytoplasmic fractions were performed to check p65^{S536} levels, β-actin and Histone H3 load control was used as fractions nuclear and cytoplasmic respectively. (**C**) IFI to see location of p65^{S536} in SCC143/E7 and SCC143/V cells. Scale bar: 5 μm.

Human Phospho-MAPK Array Coordinates

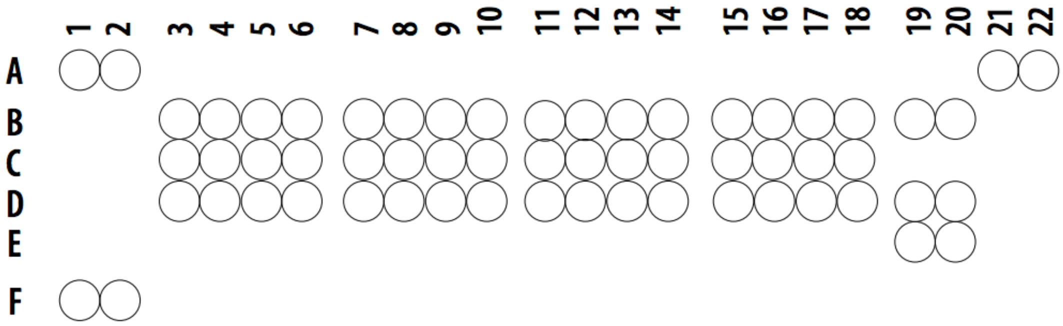

Coordinate	Target/Control	Alternate Nomenclature	Phosphorylation Site Detected
A1, A2	Reference Spots	——	——
A21, A22	Reference Spots	——	——
B3, B4	Akt1	PKBα, RACα	S473
B5, B6	Akt2	PKBβ, RACβ	S474
B7, B8	Akt3	PKBγ, RACγ	S472
B9, B10	Akt pan	——	S473, S474, S472
B11, B12	CREB	——	S133
B13, B14	ERK1	MAPK3, p44 MAPK	T202/Y204
B15, B16	ERK2	MAPK1, p42 MAPK	T185/Y187
B17, B18	GSK-3α/β	GSK3A/GSK3B	S21/S9
B19, B20	GSK-3β	GSK3B	S9
C3, C4	HSP27	HSPB1, SRP27	S78/S82
C5, C6	JNK1	MAPK8, SAPK1γ	T183/Y185
C7, C8	JNK2	MAPK9, SAPK1α	T183/Y185
C9, C10	JNK3	MAPK10, SAPK1β	T221/Y223
C11, C12	JNK pan	——	T183/Y185, T221/Y223
C13, C14	MKK3	MEK3, MAP2K3	S218/T222
C15, C16	MKK6	MEK6, MAP2K6	S207/T211
C17, C18	MSK2	RSKβ, RPS6KA4	S360
D3, D4	p38α	MAPK14, SAPK2A, CSBP1	T180/Y182
D5, D6	p38β	MAPK11, SAPK2B, p38-2	T180/Y182
D7, D8	p38δ	MAPK13, SAPK4	T180/Y182
D9, D10	p38γ	MAPK12, SAPK3, ERK6	T183/Y185
D11, D12	p53	——	S46
D13, D14	p70 S6 Kinase	S6K1, p70α, RPS6KB1	T421/S424
D15, D16	RSK1	MAPKAPK1α, RPS6KA1	S380
D17, D18	RSK2	ISPK-1, RPS6KA3	S386
D19, D20	TOR	——	S2448
E19, E20	PBS	Control (-)	——
F1, F2	Reference Spots	——	——

Figure S5. Human MAPK Pathway Array Coordinates.

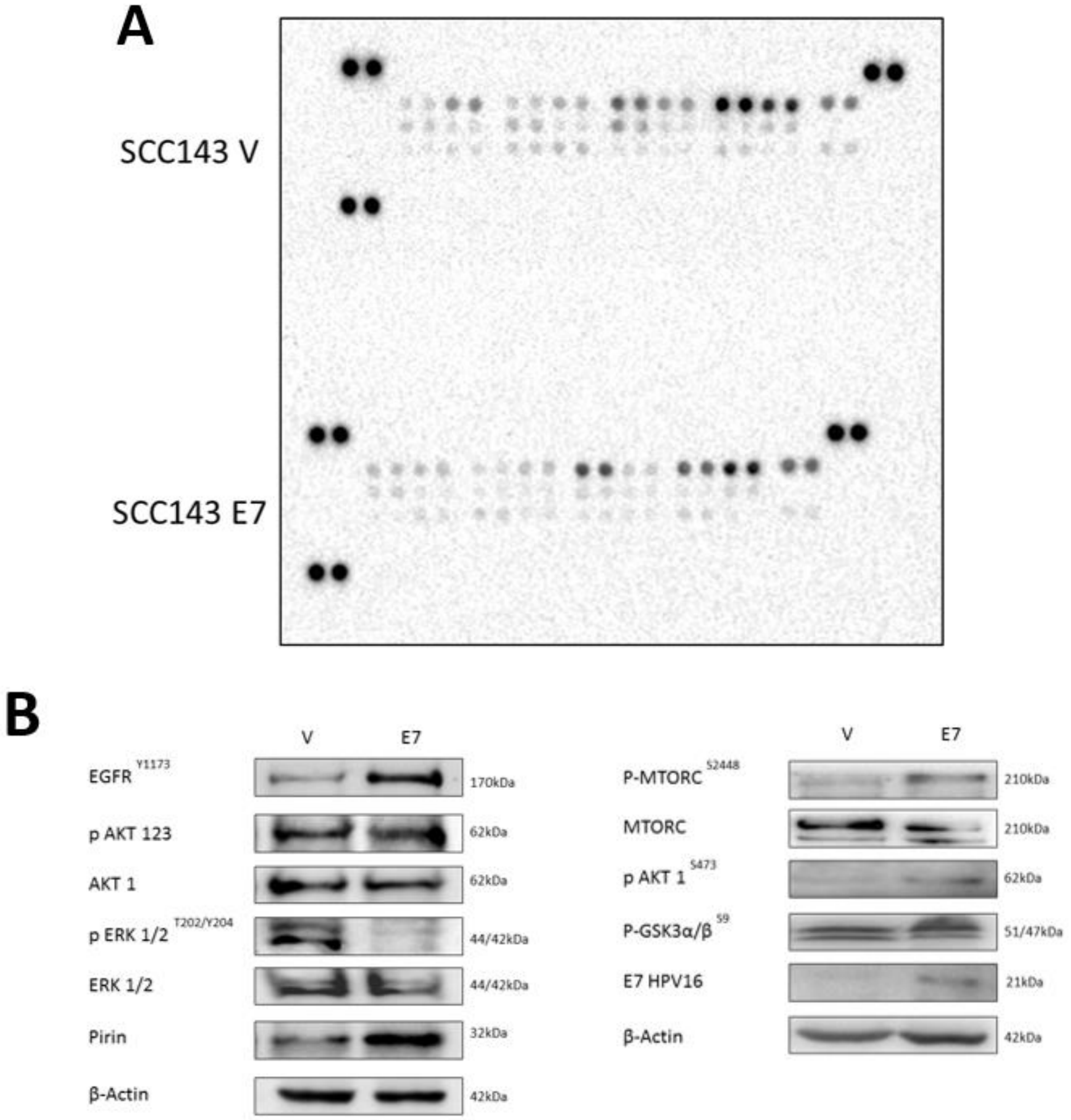

Figure S6. HPV16 E7 expression induces changes in MAPK and AKT pathways in oral cells. (**A**) Array MAPK under the conditions of V and E7 (**B**) WB were performed to analyze the levels of a pool of proteins involved with the MAPK and AKT pathways, β-actin load control was used in the cells transduced with pLXSN/V and pLXSN/E7.

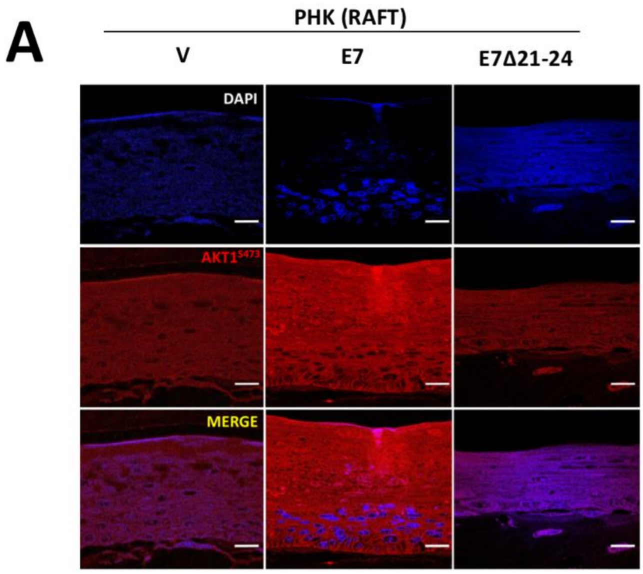

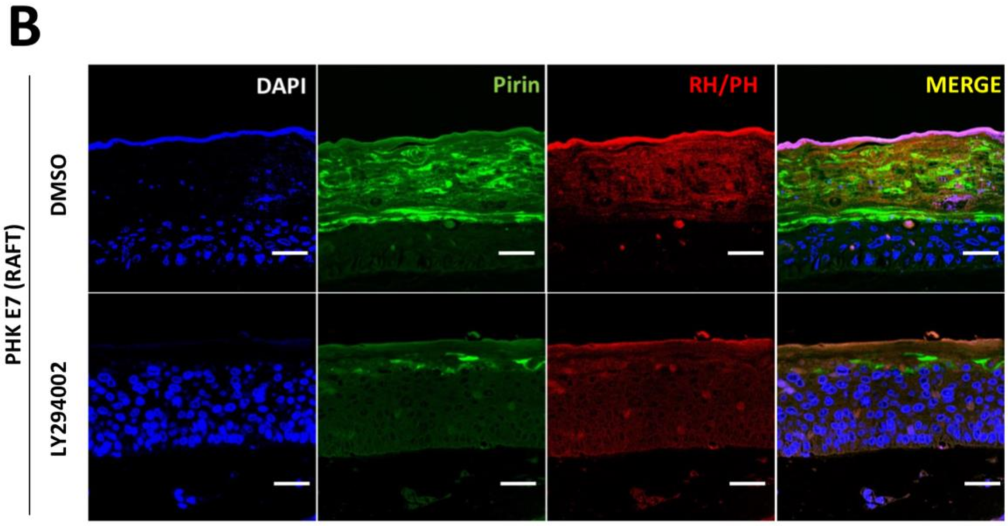

Figure S7. The binding site of pRb and the PI3K/AKT1 pathway are necessary in the regulation of AKT1 and E7-mediated Pirin expression in PHKs. Scale bar: 35 µm. (**A**) IFI reveals the increase in AKT1^{S473} in the organotypic PHK E7. (**B**) IFI to detect Pirin in PHKs treated with DMSO and LY294002. Scale bar: 35 µm.

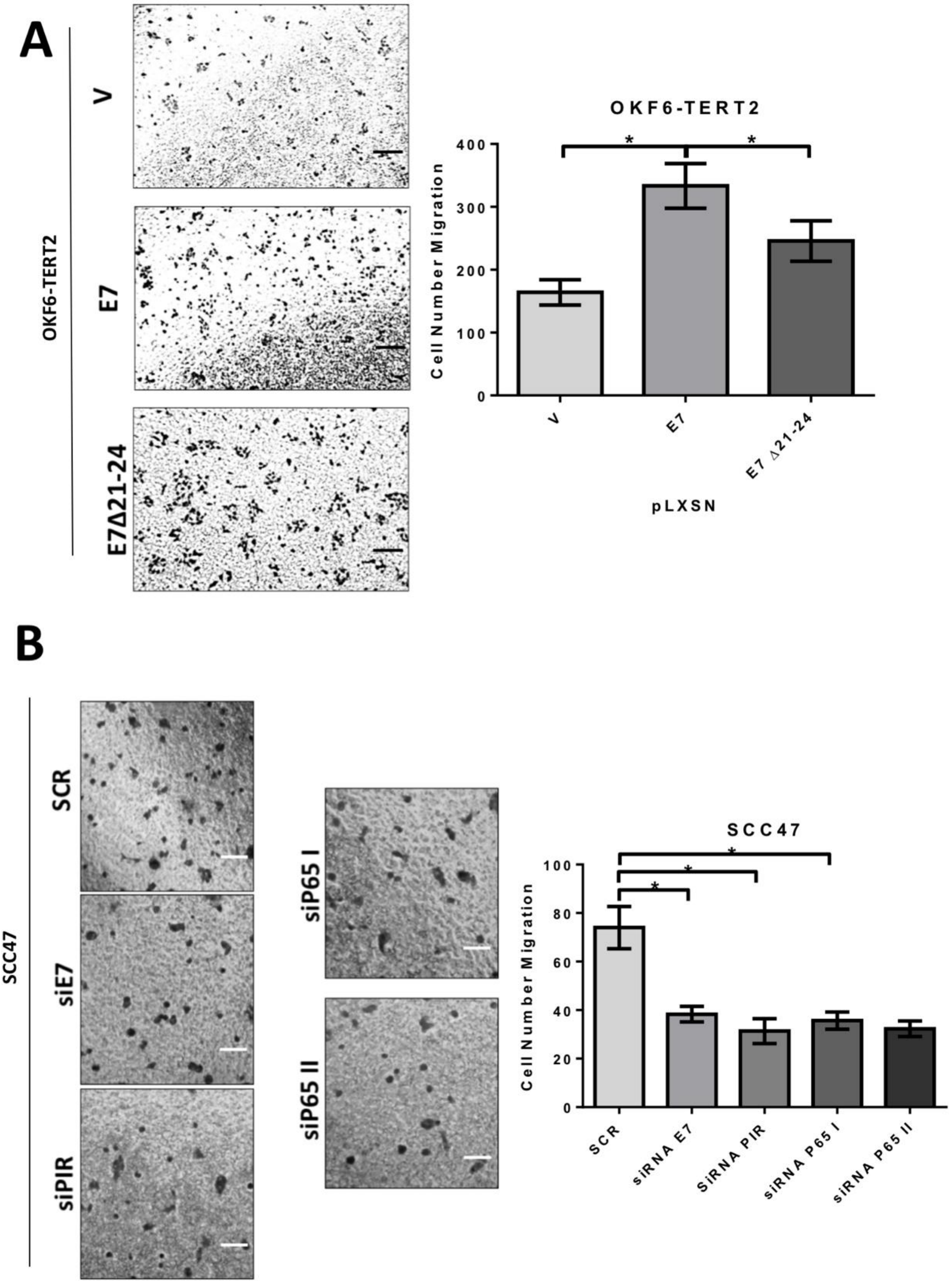

Figure S8. HPV16 E7, Pirin and p65 expression induce migration in oral cells, (**A**) Migration assay in OKF6 TERT2 E7, OKF6 TERT2 E7Δ21-24 and OKF6 TERT2 V cells was carried out for 7 h by using transwells previously treated with fibronectin. Scale bar: 40 µm. (**B**) Migration assay performed in SCC47cells previously transfected with control siRNA (SCR), siRNA PIR, siRNA P65 and HPV16 siRNA E7 was carried out for 7 h by means of transwells previously treated with fibronectin. Scale bar: 25 µm.

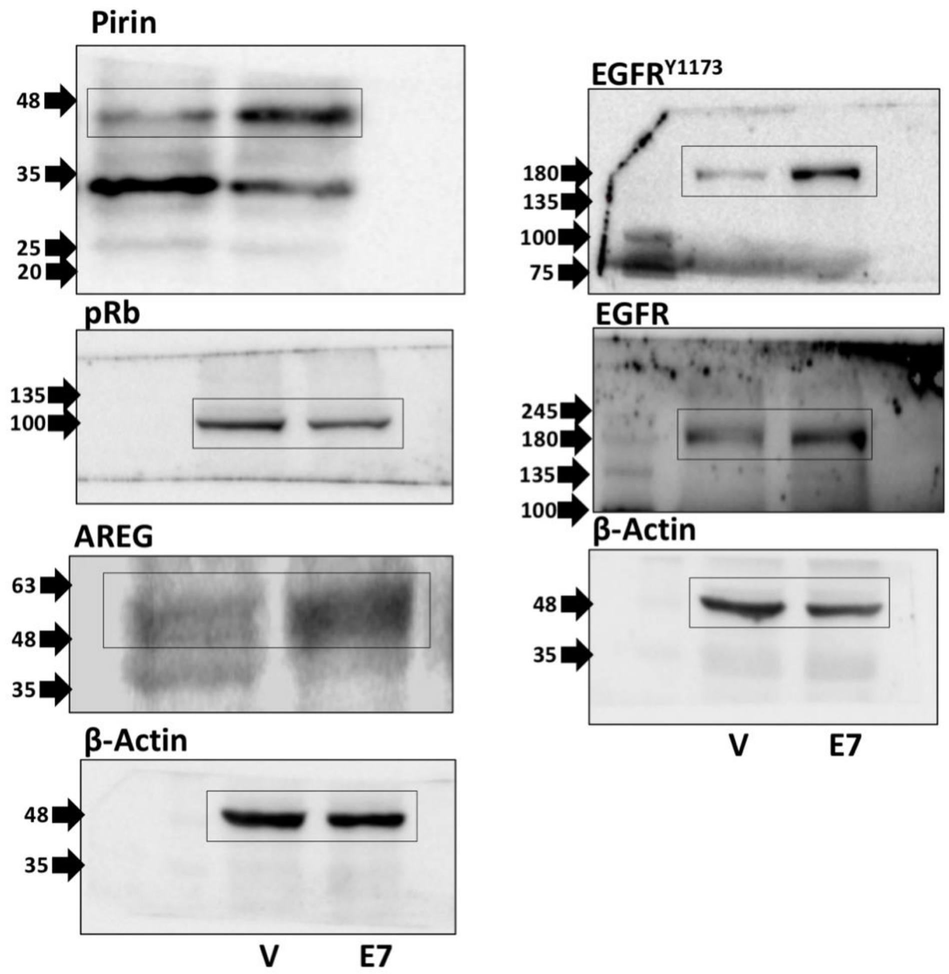

Figure S9. Unprocessed images for WB results (Pirin, EGFR, pRb, AREG and EGFR$_{Y1173}$) of SCC143 cells, corresponding to the Figures 1A, 3B and S1C.

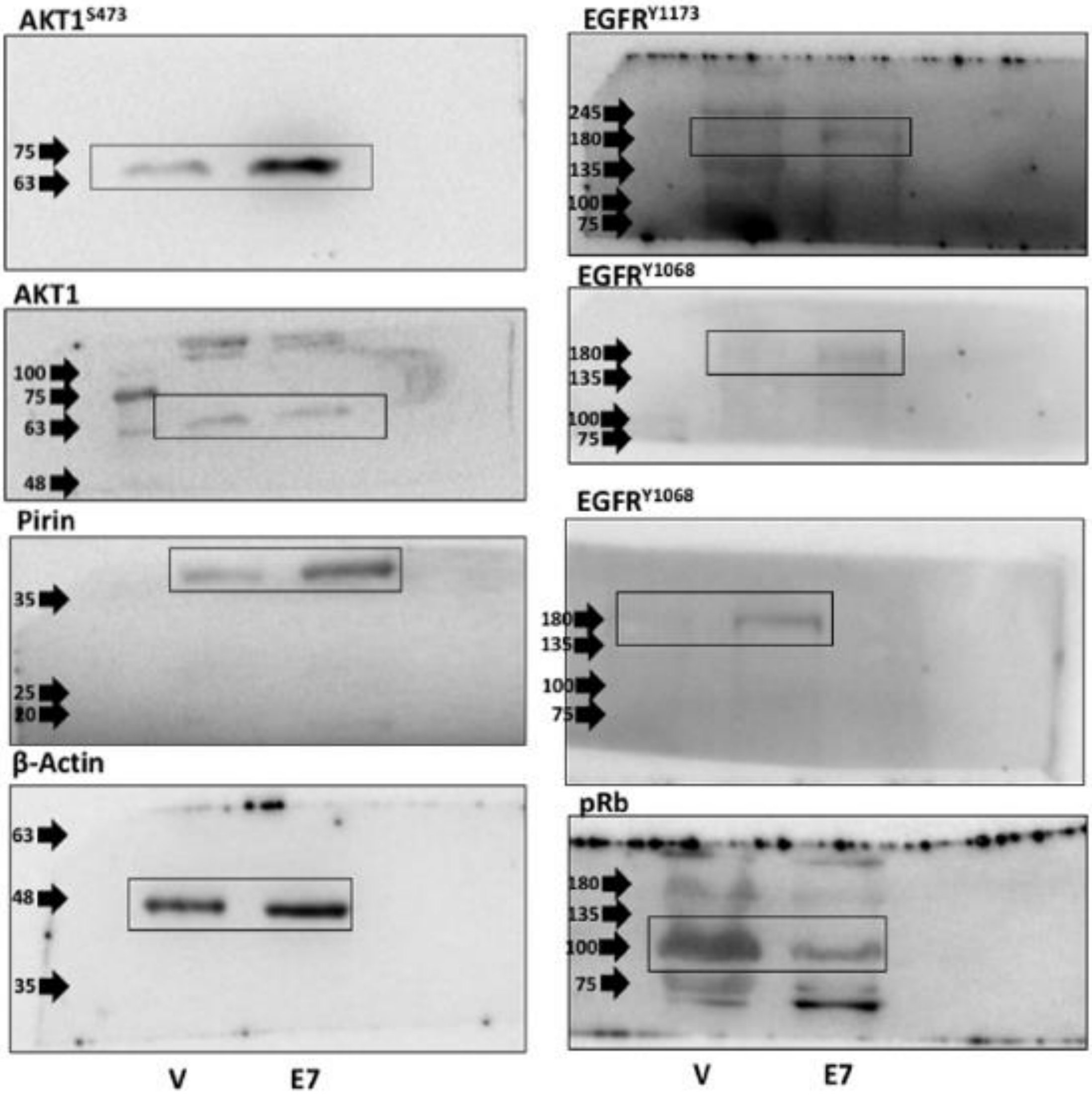

Figure S10. Unprocessed images for WB results (Pirin, EGFR, pRb, $EGFR_{Y1173}$, $EGFR_{Y1068}$, AKT1 and $AKT1_{S473}$) of OKF6-TERT2 (Raft) cells, corresponding to Figures 1F, S2A and 3C.

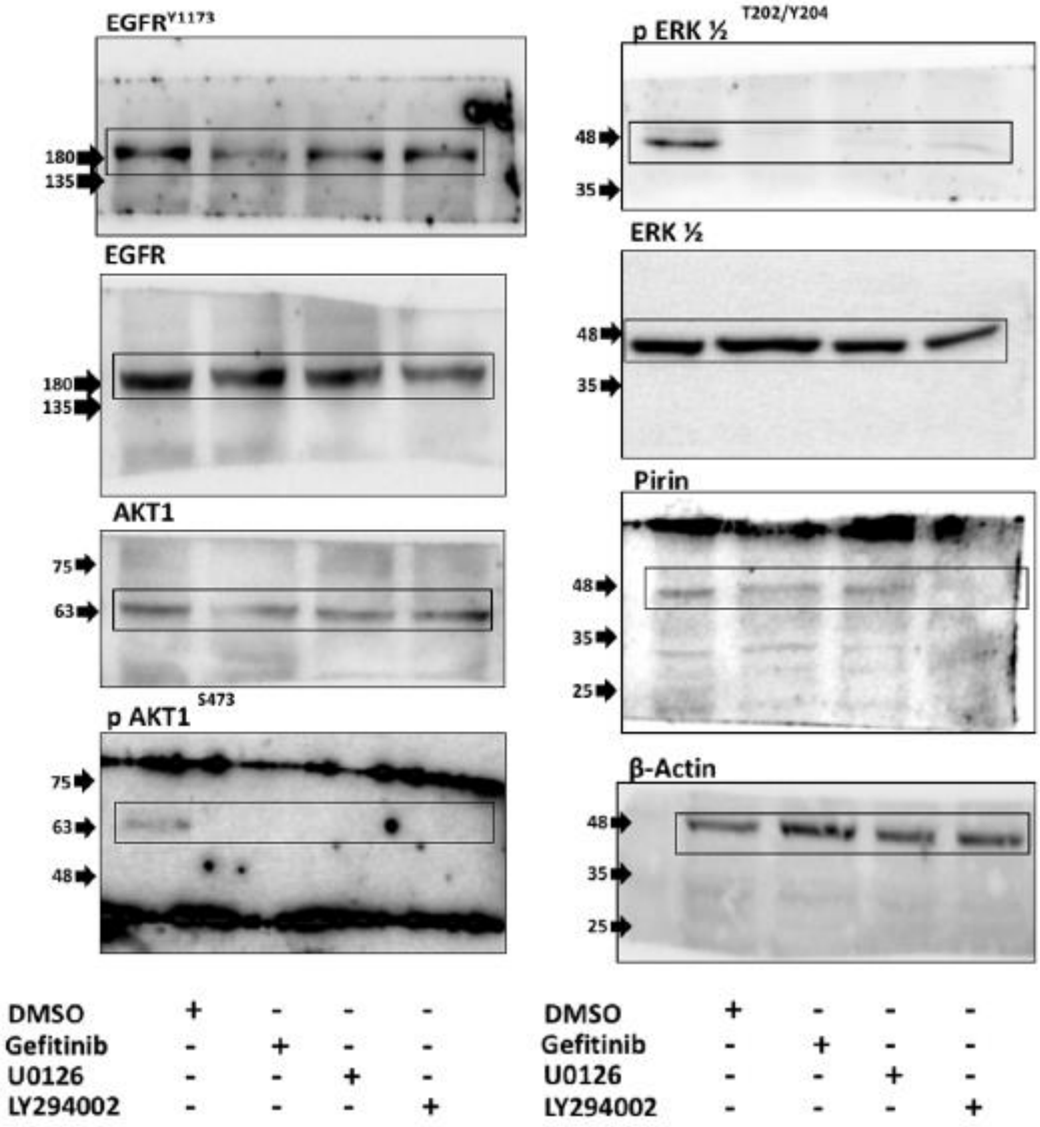

Figure S11. Unprocessed images for WB results (Pirin, EGFR, ERK1/2, p-ERK1/2$_{T202/T204}$, EGFR$_{Y1173}$, AKT1 and AKT1$_{S473}$) of SCC143 cells, corresponding to Figure 4A.

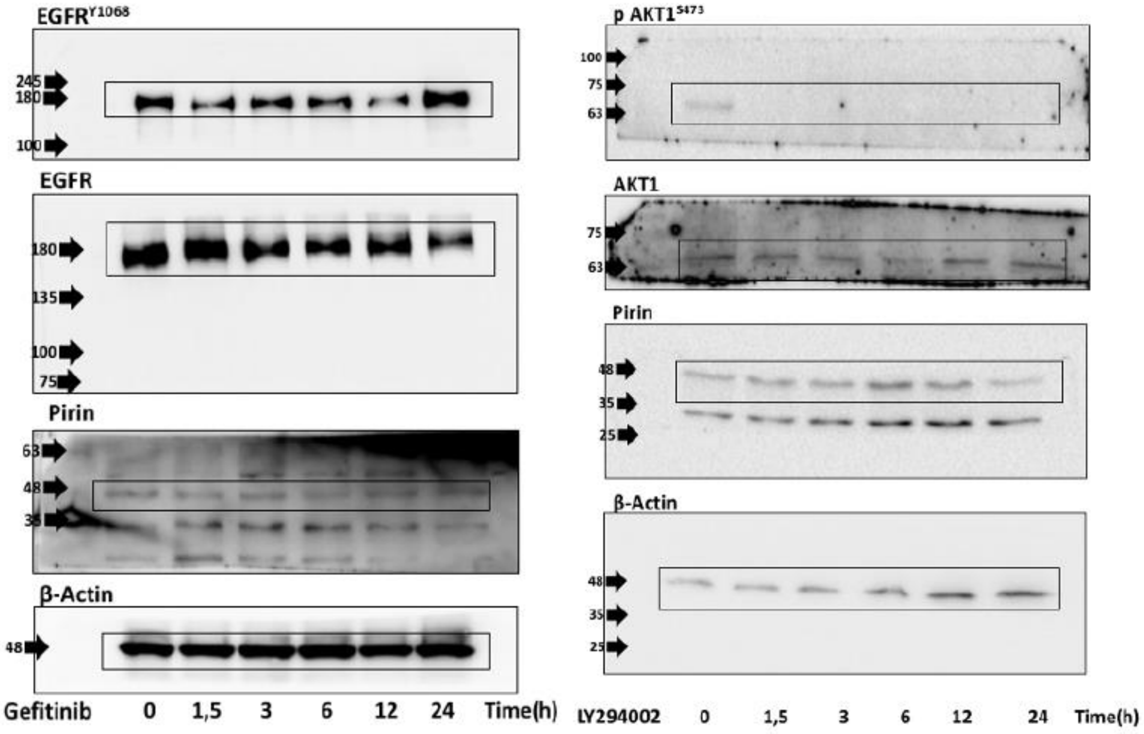

Figure S12. Unprocessed images for WB results (Pirin, EGFR, $EGFR_{Y1068}$, AKT1 and $AKT1_{S473}$) of SCC143 cells, corresponding to Figure 4B and 4C.

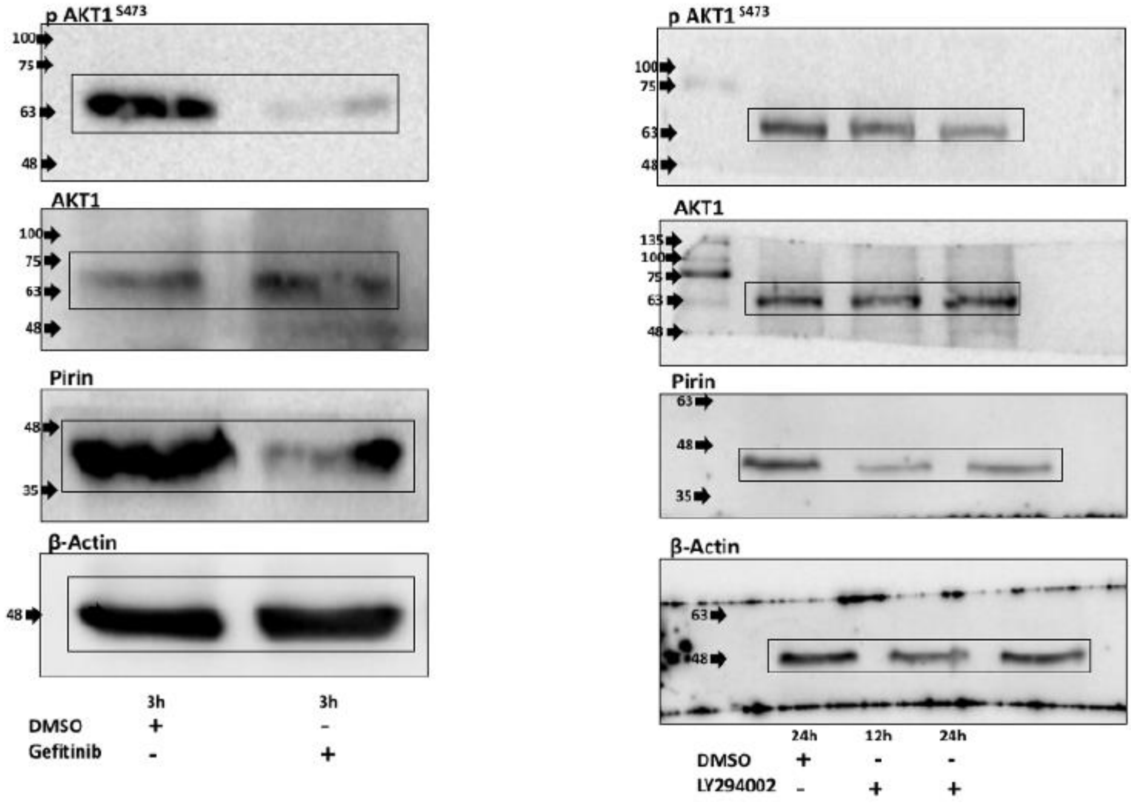

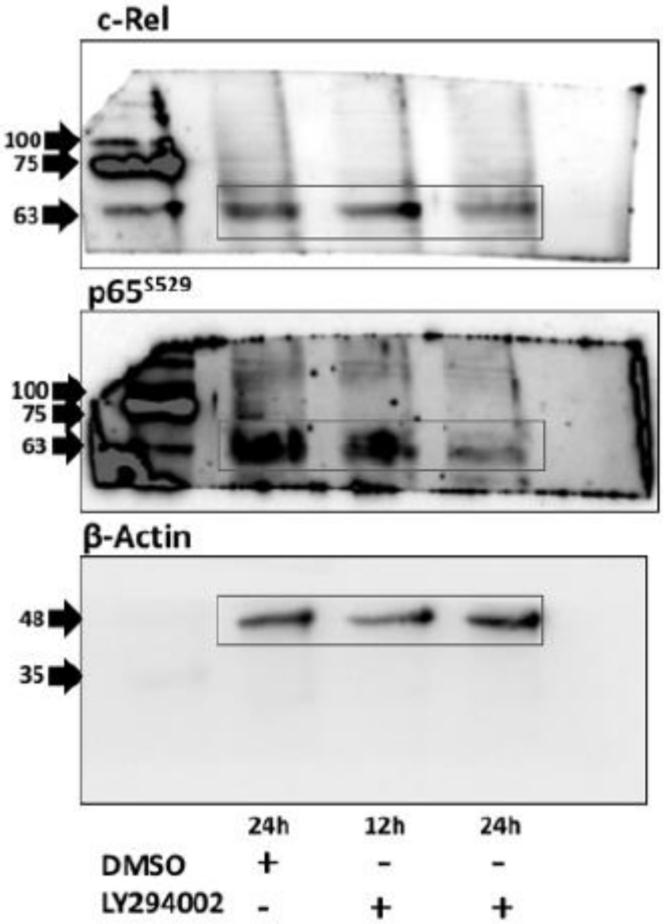

Figure S13. Unprocessed images for WB results (Pirin, c-Rel, $P65_{S529}$, AKT1 and $AKT1_{S473}$) of OKF6-TERT2 (Raft) cells corresponding to Figure 4D,E and F.

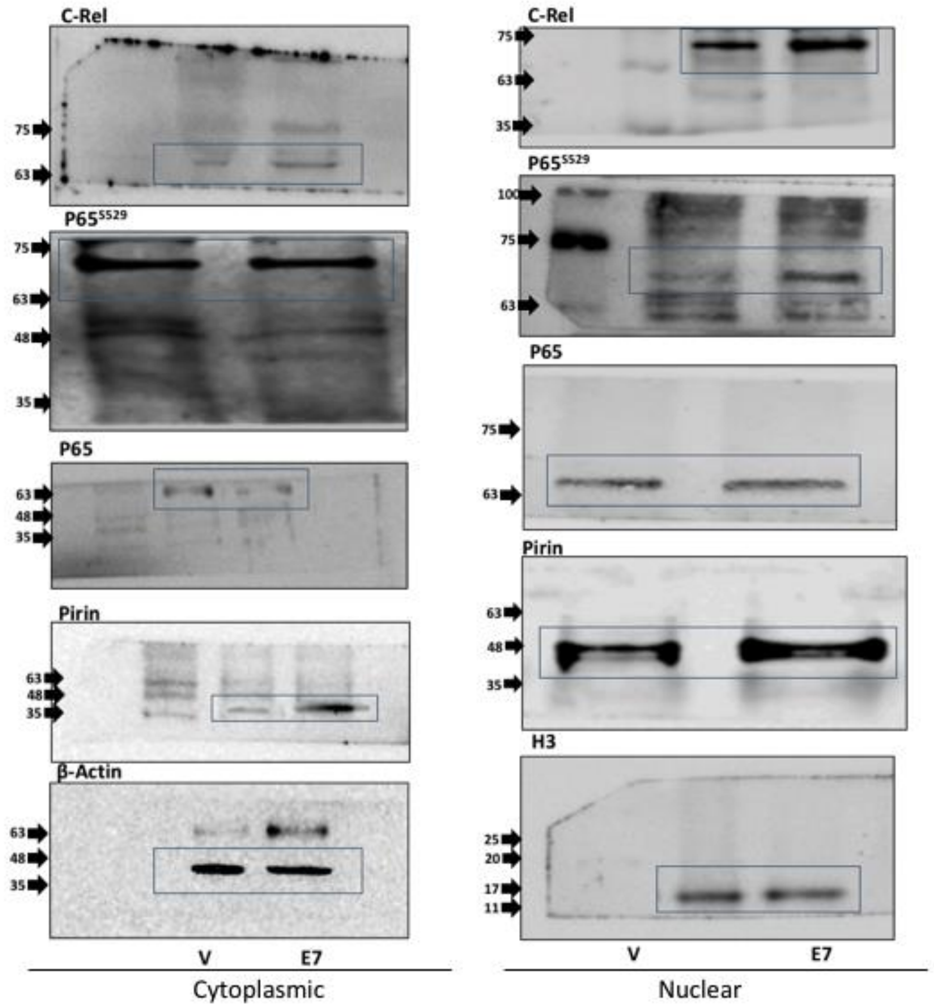

Figure S14. Unprocessed images for WB results (Pirin, c-Rel, P65$_{S529}$ and P65) corresponding to Figure 2E.

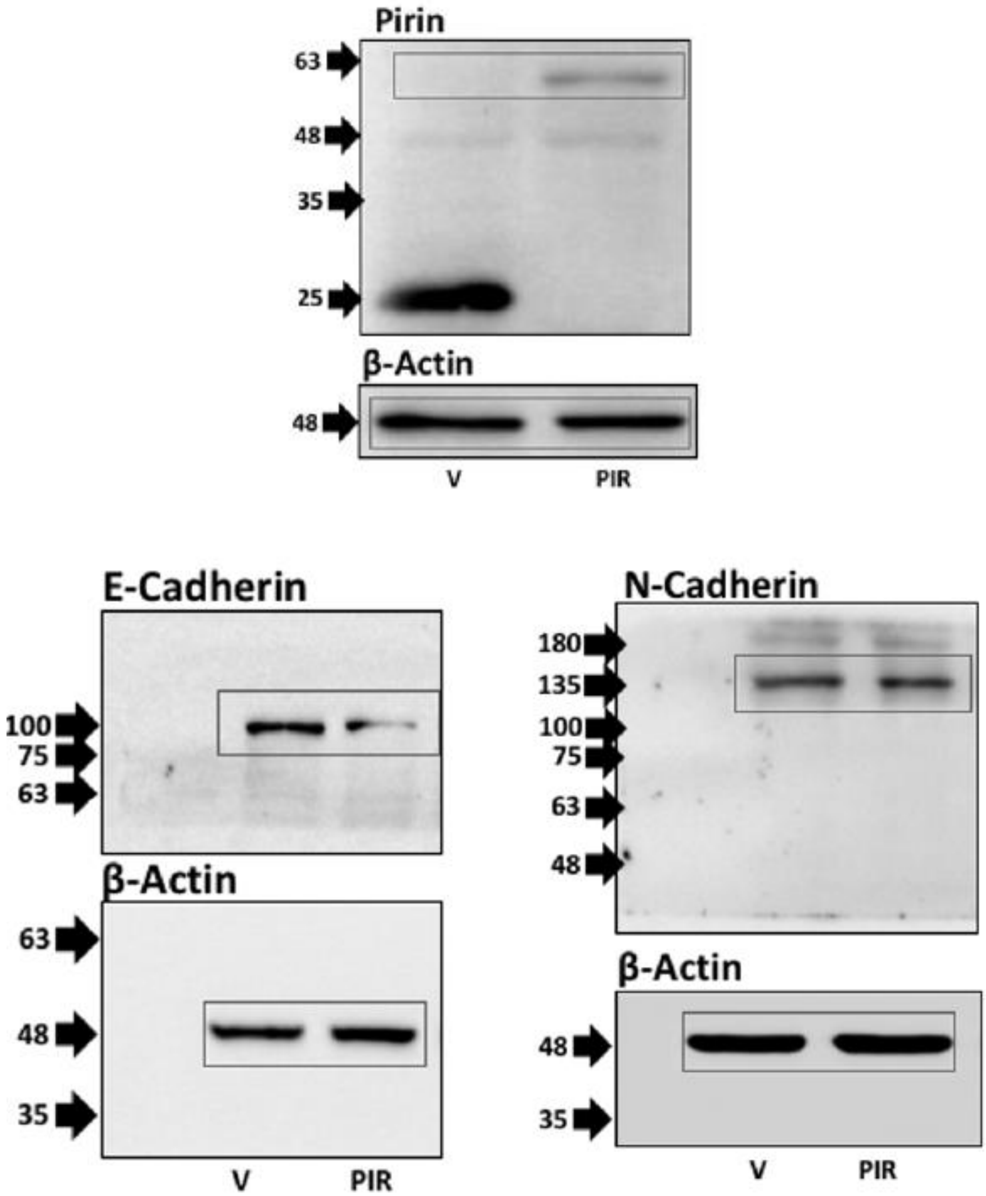

Figure S15. Unprocessed images for WB results (GFP, E-Cadherin and N-Cadherin) corresponding to Figure 6D and F.

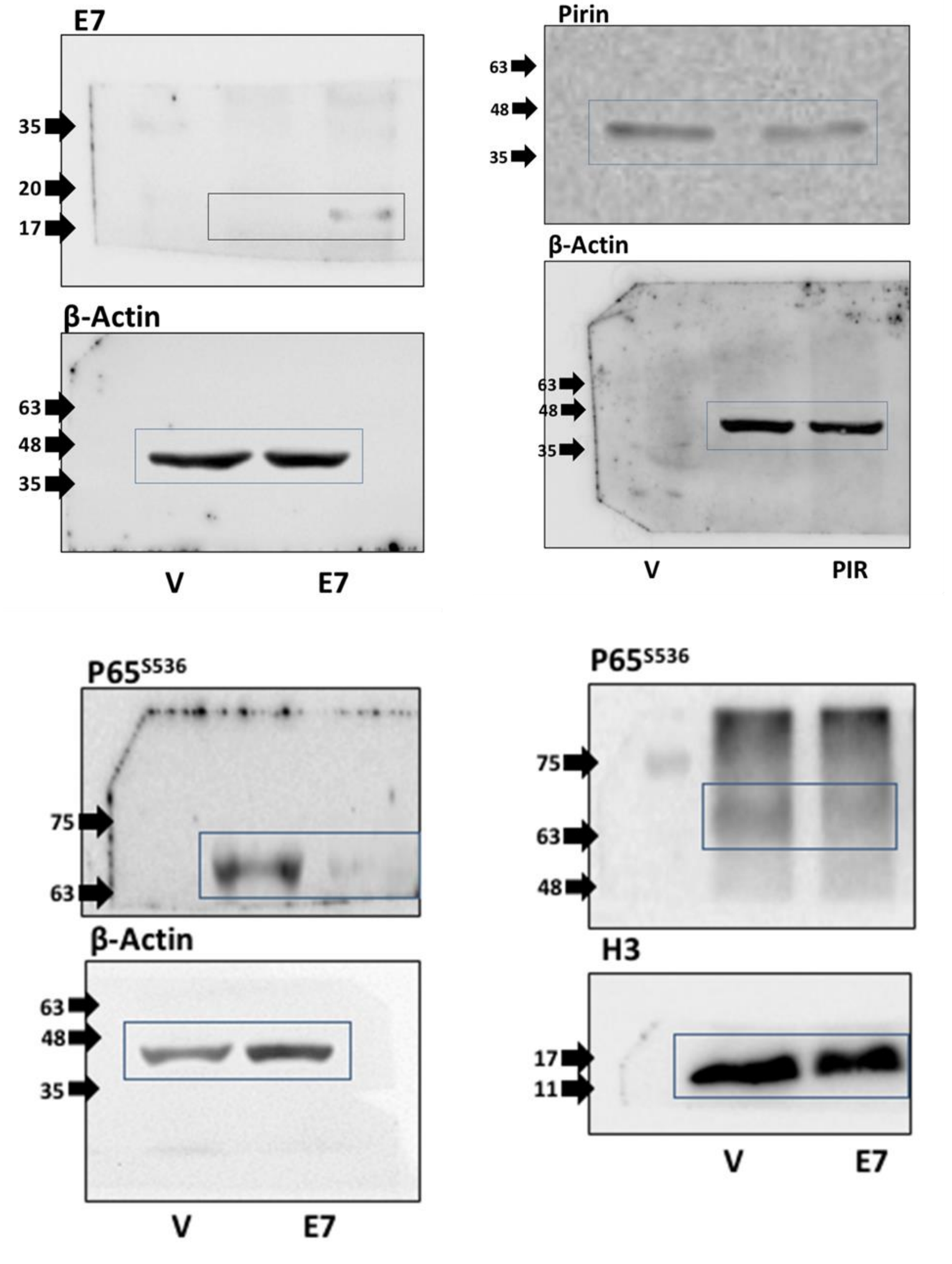

Figure S16. Unprocessed images for WB results (E7, Pirin and $P65^{S536}$) corresponding to the Figures S1A, S2B and S4B.

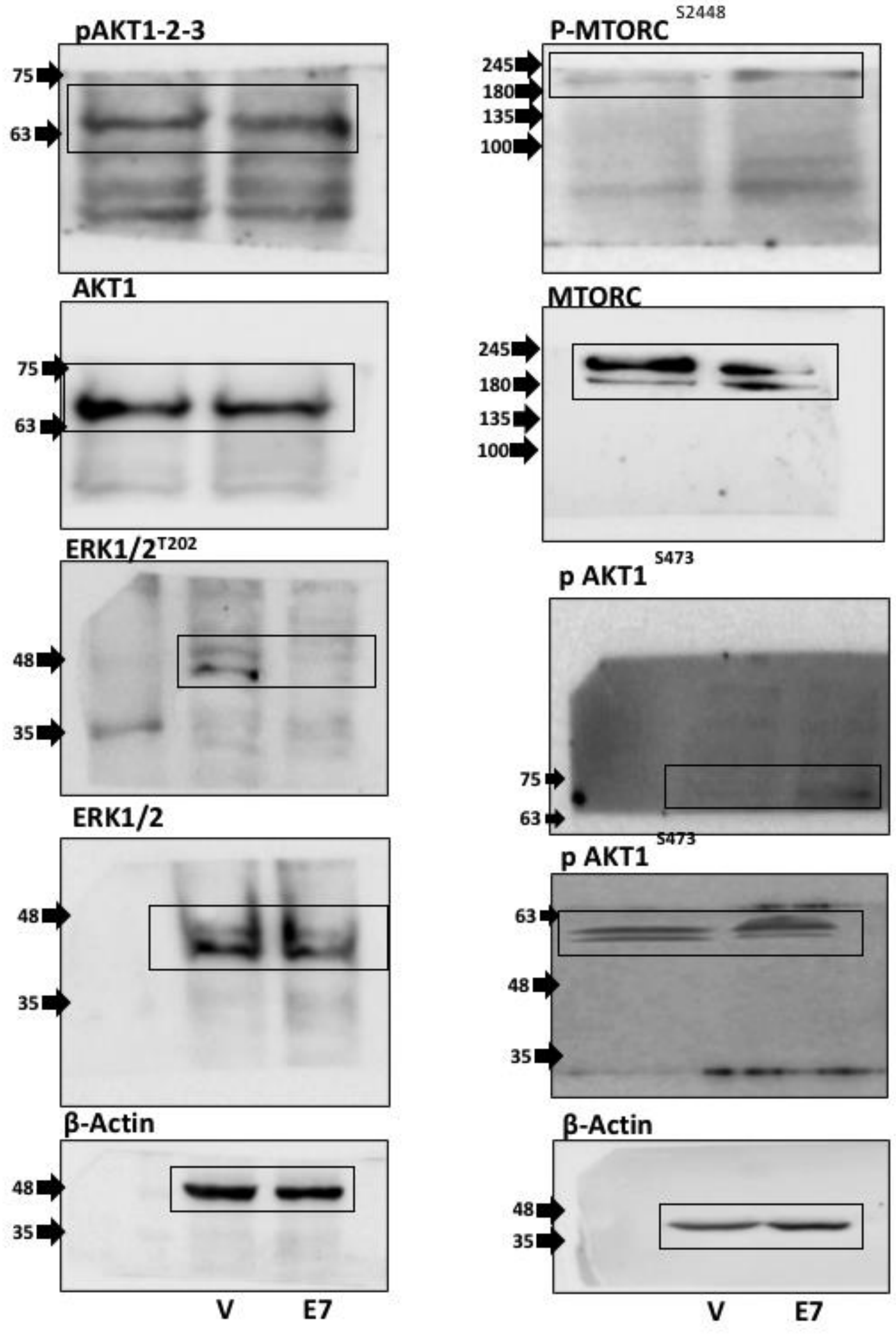

Figure S17. Unprocessed images for WB results (ERK1/2, AKT1, MTORC, pERK1/2, pMTORC, pAKT1-2-3 and pAKT1) corresponding to the Figure S6B.

© 2020 by the authors. Licensee MDPI, Basel, Switzerland. This article is an open access article distributed under the terms and conditions of the Creative Commons Attribution (CC BY) license (http://creativecommons.org/licenses/by/4.0/).
